# Lecture 4—Neuralgia

**Published:** 1873-02

**Authors:** Walter Hay

**Affiliations:** Assistant to Chair of Practical Medicine, and Lecturer on Diseases of Brain and Nervous System, Rush Med. College


ARTICLE II.—LECTURE 4—*Neuralgia.* By WALTER HAY, M.D., Assistant to Chair of Practical Medicine, and Lecturer on Diseases of Brain and Nervous System, Rush Med. College.

(Continued.)

*Gentlemen:* In my last lecture I commenced the subject of the treatment of neuralgia, which you will remember I divided into prophylactic, palliative and remedial. This classification is of course purely arbitrary, and is original with myself, and adopted as simple, and practically convenient. You will find in the course of your reading, that every author has his own ideas of classification in accordance with the leading principle which governs him in his study and management of disease. Mine has been arranged with reference to my mode of studying these diseases, and you will doubtless, in after years, make classifications for yourselves, such as observation, experience, and the natural bent of your own genius will suggest.

As I have already mentioned to you the most prominent and valuable prophylactic and palliative agents, and some of the more general curative or remedial, I will now suggest to you some of those which have been called specific or anti-toxic, and designed to counteract some special condition of the system, constituting the exciting cause of the pain. These special conditions are, more conspicuously: the malarial, rheumatic, gouty, and syphilitic.

There are three remedies which exercise a marked influence upon the malarial poison,; these are, strychnia, arsenic and quinine.

The first I have found capable of producing most valuable effects, although it would be impossible to say whether these were due to its powerful stimulant action upon nerve-tissue as a general nerve-tonic, or to any anti-malarial, or anti-toxic properties it might possess. I am inclined to believe that the former is the true reason of its beneficial effect. The only way to test this question thoroughly, would be to administer it in malarial neuralgia, in neuralgia not of malarial origin, and in some other morbid condition of the system dependent upon malarial influence, and not neuralgic in its character; and comparing the results. In the first two cases, its value cannot be disputed; in the third, it is not yet established. I know of no remedy, however, which has been in my own hands so generally useful in the treatment of neuralgia as strychnia, in doses ranging from $\frac{1}{50}$ up to $\frac{1}{16}$ grain. Below the first dose, the effect is not very decided; above the second, it is unsafe.

Arsenic has been already referred to as a valuable general nervous-tonic; its anti-malarial properties are well established likewise; it seems, therefore, to be doubly appropriate as a remedy in malarial neuralgia. In cardiac neuralgia it is especially valuable, and may be continued, as I have already told you, for many weeks, or even months.

Of all remedies applicable to the cure of malarial neuralgia, you will recognize quinine as the type. The relation of this drug to innervation is peculiar, and I believe its power over ague (intermittent fever) to be due, not to any antiseptic properties it may possess, according to some authors, nor to its power of destroying organic spores in the blood, which may or may not occasion the disease; but to its direct effect as a nerve-tonic, by which it sustains the equilibrium of functional energy, and prevents the periodical disturbances which constitute the essential factor in this form of disease. I can find no explanation of the effect of this drug; authors, as a general rule, being silent about the *modus operandi* of medicines. My own belief is, that it improves the nutrition of nerve-tissue in anæmic conditions by overcoming arterial spasm, by paralyzing the vaso-motor nerves, and thus admitting a more liberal blood-supply.

The remedy may be administered alone, or combined with iron, or with iron and strychnia.

One grain of strychnia, forty of the sulphate of quinia, dissolved in an ounce of muriated tincture of iron, and administered in doses of twenty to thirty drops, three or four times daily, will suffice for any ordinary case of this form of disease.

Authors speak of this form of disease as being very rare. It is so, doubtless, in the large cities in high latitudes, in which most of them have acquired their experience; but in lower latitudes and warmer climates it is of very frequent occurrence as a sequel or complication of malarial fevers.

Colchicum has been used freely in the treatment of neuralgia, and, where the disease seems to have its origin in the dyscrasia of gout or rheumatism, is valuable. This complication is, I think, more infrequent than is generally believed. Many cases of what was formerly called rheumatism, are now, under more accurate methods of diagnosis, clearly identified as neuralgic.

We come now to that much-abused remedy, "mercury." From what I have already said of the universality of debility as an essential element in neuralgia, it would seem that mercury, the type of analeptic, of tissue-destroyers, could find no place whatever among its therapeutic category, without trenching upon the domain of the little-pill gentry, under their motto "*similia similibus.*" Nevertheless, in certain forms of the disease which have the syphilitic diathesis as their exciting cause, this agent is invaluable, and as these cases are by no means rare, the remedy is often called into play. For you must remember that in the human economy, as in all other organisms, repair, reconstruction, necessarily implies waste, destruction; new tissue cannot grow, new cells cannot germinate, until the old are destroyed and removed to make room for them.

Of the preparations of mercury, the chlorides, iodides, and bromides, are the most useful. The bichlorid, from its greater solubility, and consequent susceptibility of administration in very small doses, *i. e.*, from $\frac{1}{40}$ of a grain up to $\frac{1}{10}$, is better adapted to this class of cases than calomel. The same rule should apply to the double salts, with iodine and bromine. But they have been found to be so irritating to the stomach that they have not come into general use. The proto-iodid and proto-bromid seem best adapted to the syphilitic diathesis, when it becomes an efficient cause of neuralgia. I believe that I was the first to use the proto-bromid, at any rate in this part of the world. When I first began to experiment with it, it was a rare chemical, prepared only by Merk, of Darmstadt. The worst case of sciatica I ever saw, which had baffled the skill of some of the best physicians in the United States, during seven years, after having resisted the influence of every remedy, regular and irregular, (they were all defective,) yielded at last to this. The patient was a man 60 years of age, of almost gigantic size, who had been, during the four preceding years, attacked about the first of January of each year, and about six or seven o'clock in the evening of each day, with sciatic neuralgia, of the most intense severity. I have known him to stand during the whole of fourteen successive nights, holding on to the bedpost screaming with pain; during the day he would attend to his business (he was a merchant) just as usual. I have kept him under the influence of chloroform many entire nights, without any permanent relief. Discovering accidentally, after three years of experimenting, a copper-colored scar with irregular margins, a little above the external malleolus, I learned from the patient that it was the scar of an old ulcer that had troubled him for many years, resisting all sorts of treatment, until it had healed spontaneously, as he said, after his recovery from an attack of cholera, during which he had taken large doses of calomel. Acting upon this hint, I gave him bromid of mercury in $\frac{1}{4}$ grain doses; and the sciatica disappeared, and did not reappear during the remaining ten years of his life, which was terminated by phthisis at 70 years. The neuralgia in this case was doubtless due to thickening of the periosteum in the posterior portion of the sciatic foramen; the case had previously been benefited temporarily by blisters.

The next remedy to which I shall call your attention is iodide of potassium. This seems peculiarly adapted to neuralgias of syphilitic origin, and should be given pretty freely, in doses of ten grains or more, every four or six hours.

I have recently treated, successfully, a case of trigeminal neuralgia of twenty years standing, resulting from an injury to the head consequent upon a railroad accident, with ten grain doses of iodide of potassium, after every other treatment had been abandoned as useless.

There was probably in this case either a thickening of the internal periosteum of the skull, or an hypertrophy of the connective tissue of the nerves themselves.

Cannabis or Indian hemp, or as sometimes called, haschish, is one of the most useful of the narcotics in the treatment of this disease. Much disappointment is experienced in its use, in consequence of the variable strength of the preparations usually found in the shops. When the extract, in which form it is most conveniently used, is made from the leaves of the plant grown in a tropical climate, it may be depended upon when administered in one grain doses every six or eight hours, to allay pain and produce sleep. When prepared from plants of the cannabis indica, grown in this country or in Europe, it has no value whatever.

A class of remedies has recently come into general use, of which bromine is the active principle. In combination with potassium and sodium, calcium, lithium, mercury and iron, in the forms of bromides of those substances respectively, these agents seem to possess peculiar value in the treatment of neuralgia, into which congestion, hyperæmia of the nerve-centres, enters as a factor; and these are many. Hyperæmia is not by any means inconsistent with the idea of debility, indeed it is a very frequent accompaniment, being itself a local expression of vaso-motor debility exhaustion. Indeed, within my own experience, they constitute the best prophylactics against this form of neuralgia. In trigeminal neuralgia, sick headache, I believe the bromide of sodium to be the most valuable agent that has thus far come under my own observation. I prefer it to the bromid of potass. for two reasons, which have nothing whatever to do with its therapeutic effects. The first of these, is its less disgreeable taste. The second, that it does not, like the bromid of potass., produce a troublesome cutaneous eruption.

The next remedy which I shall mention to you as useful in the treatment of neuralgia, is the actual cautery. The remedy is old; has been abandoned and revived. The cauterizing iron should be applied at a white heat, in order that an eschar may be formed without suppuration. I can speak very superficially of the use of this remedy; it is one which promises much, from the profound alteration which it must induce in the nutrition of the tissues in the neighborhood of the point of application. You will find it difficult to induce patients in private practice to submit to this remedy, although it is not so painful perhaps as many now in use. It is applied now by means of a galvanic battery. Bunsen's is the best, and is thus termed the galvano-cautery.

Moxas have been applied to cure neuralgia, more especially in the form of sciatica. I think "the game is hardly worth the candle," as it would be difficult to say which was the lesser of the two evils, or whether the remedy was not worse than the disease.

Electricity, as a remedy for neuralgia, presents much that is attractive to the theorist, while at the same time its practical value is endorsed by some of the highest authorities. As for example: Messrs. Althaus and Anstie, in England; Duchenne and DuBois Raymond, in France; and Remak and Moritz Meyer, in Germany.

Attempts have been made to prove the identity of electricity and nerve-force; these attempts have thus far proved failures. They may be identical, but it has not yet been demonstrated. Should it be identical with nerve-force, then in it we must have the theoretical remedy for every perturbation of innervation; it would be a nervous panacea, and such you would believe it to be already, if you swallowed without any grains of allowance all that its enthusiastic advocates claim for it. But I must warn you, that valuable as it is, the application of electricity is purely empirical. Why it should benefit in some cases, and injure in others, is not satisfactorily explained as yet.

Systematic writers all tell us that the interrupted current will produce this or that effect, and the constant current this or that other effect, but fail to tell us how these effects are produced, and why; and, moreover, are sadly at variance as to their statements of facts.

The whole subject of electrical therapeutics presents a wide field for examination and experiment.

With regard to its application in neuralgia, the preponderance of evidence seems to give the preference to the continuous current over the interrupted, and this agrees with my own observations and experiments, which have not, however, been very numerous. With the interrupted, or, as it is sometimes called, the Faradaic, current, I have effected little.

The continuous or constant current, or primary current, as it is called, has produced astonishing results in the hands of Meyer and Remak. It should be applied only a few minutes at a time, daily, and repeated for many days. Sometimes, however, the disease is arrested after two or three applications; sometimes not until after fifty or sixty.

With regard to the direction of the current, it seems to be of little consequence, so long as the current passes through the diseased nerve or centre, according to some authors. Upon this subject there is much difference of opinion expressed by systematic writers on electro-therapeutics, some specifying that a direct, others that an inverse current should be used.

DuBois Raymond gave the name electrotonus to the condition of a nerve within the circuit of a constant battery. He found, what one would infer reasonably, that the original, or what he calls the inherent nerve-current, was increased in power when the artificial current was in the same direction, and diminished when it passed through the nerve in an opposite direction. When the nerve current is increased, it is said to be in the *positive phase;* when it is decreased, it is said to be in the *negative phase.* The increase takes place when the positive electrode (or anode) is nearest the transverse section, (the experiments were made on segments of nerves); the decrease when the negative electrode (or cathode) is nearest.

Brenner, a German electrician, says, that it is rarely possible to conduct the galvanic current in the direction of a certain nerve-current, because the poles are almost always placed on points not having equal physiological importance, and hence the relative positions of the poles are to be considered as indicators of the physiological effects of the current. His method, which is confirmed by Pfluger, and which seems to be the most in harmony with what we know of the relations of electricity to nerve-force, is, to give that pole whose action is most appropriate to the case, the position most favorable, in regard to conduction, for its working on the nerve.

Mr. Althaus, the highest English authority upon electro-therapeutics, says, that, whether the application be central or peripheral, the positive pole alone should be applied to the part which we wish to affect. Eulenburg, a distinguished German electrician, agrees with him; and further states that the negative pole placed in this position will do damage.

Mr. Anstie and Reynolds declare, that while theoretically direct and inverse currents should have different effects, practically they do not, either in the case of spasm or pain.

Dr. Buzzard reported a case of cervico-brachial neuralgia, in which the same effect was produced by placing the positive pole upon the nape of the neck, and the negative in the hand, or *vice versa*.

These differences of opinion amongst the very highest authorities, will demonstrate to you the truth of what I said at the beginning of my remarks upon this subject, *i. e.*, that the application of electricity as a therapeutic agent in the treatment of nervous diseases is yet empirical. To establish laws for its rational and intelligent application more experiments and more facts are demanded.

It is necessary that these experiments should be made, and these facts be collated by you, gentlemen, and that the use of this valuable and powerful agent should not be abandoned to charlatans.

The first electrical machines used in therapeutics, were those of nature's manufacture. It is stated that one thousand years ago the natives of South Africa placed their sick children in a hole filled with water, in which were placed torpedos, one of the cartilaginous fishes, having a portable battery always ready for use. A similar treatment for gout was used by Scribonius Largus, a physician who lived in the time of Tiberius Cæsar. Pliny and Dioscorides both refer to it. The modern application of this method dates back about one hundred years, since the report to the *Societe Royale de Medecine* (Paris), by Mauduyt, in 1773.

